# Normative data of the Italian Famous Face Test

**DOI:** 10.1038/s41598-024-66252-1

**Published:** 2024-07-03

**Authors:** Martina Ventura, Alessandro Oronzo Caffò, Valerio Manippa, Davide Rivolta

**Affiliations:** 1 The MARCS Institute for Brain, Behaviour, and Development, Western Sydney University, Sydney, Australia; 2 Department of Education, Psychology and Communication, University of Bari Aldo Moro, Bari, Italy

**Keywords:** Famous face, Face recognition, Metacognition, Social cognition, Neuroscience, Psychology, Neurology

## Abstract

The faces we see in daily life exist on a continuum of familiarity, ranging from personally familiar to famous to unfamiliar faces. Thus, when assessing face recognition abilities, adequate evaluation measures should be employed to discriminate between each of these processes and their relative impairments. We here developed the Italian Famous Face Test (IT-FFT), a novel assessment tool for famous face recognition in typical and clinical populations. Normative data on a large sample (N = 436) of Italian individuals were collected, assessing both familiarity (*d′*) and recognition accuracy. Furthermore, this study explored whether individuals possess insights into their overall face recognition skills by correlating the Prosopagnosia Index-20 (PI-20) with the IT-FFT; a negative correlation between these measures suggests that people have a moderate insight into their face recognition skills. Overall, our study provides the first online-based Italian test for famous faces (IT-FFT), a test that could be used alongside other standard tests of face recognition because it complements them by evaluating real-world face familiarity, providing a more comprehensive assessment of face recognition abilities. Testing different aspects of face recognition is crucial for understanding both typical and atypical face recognition.

## Introduction

Face processing is critical for social interaction, as it conveys various information, such as identity, emotions, sex, age, attractiveness, and intentions^1^. Face recognition, in particular, is pivotal since it allows the engagement with behavior, intent, and suitable social responses^2^. Conversely, failures in face identification may hinder the ability to draw upon past social contacts for guiding appropriate role-based interactions^3,4^. Indeed, proficiency in face recognition is considered predictive of social abilities^5,6^, while deficits in face processing are associated with social inhibition and anxiety^5,7^.

Face processing encompasses three main dimensions: (i) *face perception*, i.e., the ability to perceptually judge whether two pictures represent the same or different people, (ii) *face memory* i.e., the ability to learn and recognize, after a variable time-interval, unfamiliar faces, and (iii) *face recognition*, i.e., the long-term ability to recognize familiar and/or famous faces^8,9^. Although all these three components are critical for typical social cognition, face perception, familiar and unfamiliar face recognition, as well as the different kinds of familiar faces (e.g., partner’s face; famous faces; experimentally learned faces) seem to be based on partially different and independent cognitive and neural processes^10,11^. This dissociation implies that patterns of face processing in typical and clinical populations could vary among different people.

Given the significance and complexity of human face processing, it's crucial to utilize effective evaluation measures to discern each of these processes and their associated skills. However, while several standardized tasks have been developed for face perception (e.g., Cambridge Face Perception Test^12^) and memory (e.g., Cambridge Face Memory Test^13^), only a few reliable and widely accepted famous face tests have been created, with the main limitation to be country-specific, age-specific, and, sometimes, paper-based^14–16^. To our knowledge, there is only one famous face test validated on an Italian sample^15^, but stimuli selection and data collection occurred more than 20 years ago, a long span for such a time-dependent test. The accurate assessment of face recognition is especially important in the context of various psychiatric and neurological conditions, such as Alzheimer’s disease, schizophrenia, and some neurodevelopmental conditions such as prosopagnosia and autistic spectrum disorder^17–22^, the latter showing social skills impairments along with serious face processing difficulties^23^.

It is now acknowledged that some conditions may show difficulties in different—but not all—aspects of face processing, for example, limited to retrieving semantic information about a specific person, but they can tell whether they had previously seen that face^24^, or the other way around^25^; others may present impairments in face recognition but not perception^26^, and others can have difficulties with newly learned faces but not highly familiar faces (e.g., friends or famous people)^16^ This latter aspect has been proved for prosopagnosia, a condition characterized by severe difficulties in face identity processing without associated social or cognitive deficits ^27^, suggesting the existence of different subtypes associated with different difficulties and social implications^28^. It is possible that a dissociation between different aspects of face processing could also be present in autism spectrum disorder patients or other clinical populations.

However, studies on face recognition usually employed experimental familiarization techniques with initially unfamiliar face stimuli (e.g., CFMT); here, participants need to recall and subsequently recognize faces they had never seen before, which means their representations can only comprise one, or few very brief exposures. In this case, thus, short-term, unfamiliar face memory was mostly assessed^16^. A great deal of evidence showed that familiar and unfamiliar faces are associated with separate cognitive representations^29^. Familiar faces refer to those identities we have seen frequently across diverse contexts, encompassing various expressions, viewing angles, and lighting conditions. Consequently, mental representations of familiar faces are comprehensive, drawing from a variety of experiences to form a stable impression of a particular individual (as in the case of famous faces), which differs from unfamiliar ones, as mentioned above^9,30^.

Some theories suggest that also metacognitive abilities can affect cognitive performance^31^; that is, if people believe to have poor memory skills, they tend to poorly perform on actual memory tasks. Consistently, recent studies indicate that super-recognizers—individuals with exceptional face-processing abilities compared to the average population—demonstrate not only higher accuracy and greater confidence in their face-recognition ability^32^ but also a self-awareness regarding their superior capabilities^33^. This prompts a broader question of whether people displaying different levels of performance also show varying degrees of insight into their own performance and competence. So far, results on the general population are mixed^34^.

Thus, our study aimed to (i) develop a new test for famous face recognition (the Italian Famous Face Test—IT-FFT) and collect normative data on a large sample of Italians; (ii) ascertain whether individuals have insights into their overall face identification skills.

## Methods

### Participants

Four-hundred thirty-six (436) Italian participants (321 F) were included in this study (Age range: 18–60 years; *M*_age_: 28.60 years; *SD*_age_: 11.14 years) via snowball sampling. All participants provided informed consent before completing the experiment. Exclusion criteria were history of neurological diseases, cerebral stroke, epilepsy or epileptic seizures, head injury with loss of consciousness, severe medical conditions or psychiatric disorders, and alcohol or drug. All participants had normal or corrected-to-normal vision. The study was approved by the Ethical Committee of the Institution and was performed following the Helsinki Declaration and its later amendments.

### Tasks

#### The Italian Famous Face Test

The Italian Famous Face Test (IT-FFT) comprises 100 images, 50 depicting famous and 50 unknown people. A total of 23 stimuli depicted faces of Italian celebrities, while the remaining (N = 27) showed foreign celebrities. The famous faces span various professional categories—actors, singers, politicians, scientists, and athletes—encompassing diverse historical periods. This deliberate selection aimed for a balanced representation of Italian and international fame, while also accounting for potential age-related differences among participants. Both photos of famous and non-famous people were sourced from the web, ensuring that the chosen pictures closely match the ethnicity, sex, and age within the two categories. The selected photographs for each distinct persona exhibited a facial expression deliberately kept as neutral as possible. Given that people with face recognition difficulties (e.g., prosopagnosia) often report taking advantage of external features (e.g., eye colors, hairstyle) to recognize people^35^, all the images were cropped to an oval shape and displayed in black and white to exclude most of the faces’ characteristics which could serve as a cue to recognition. All the celebrities included in our test are listed in a data repository (10.6084/m9.figshare.25603887).

#### Procedure

**Figure 1:**
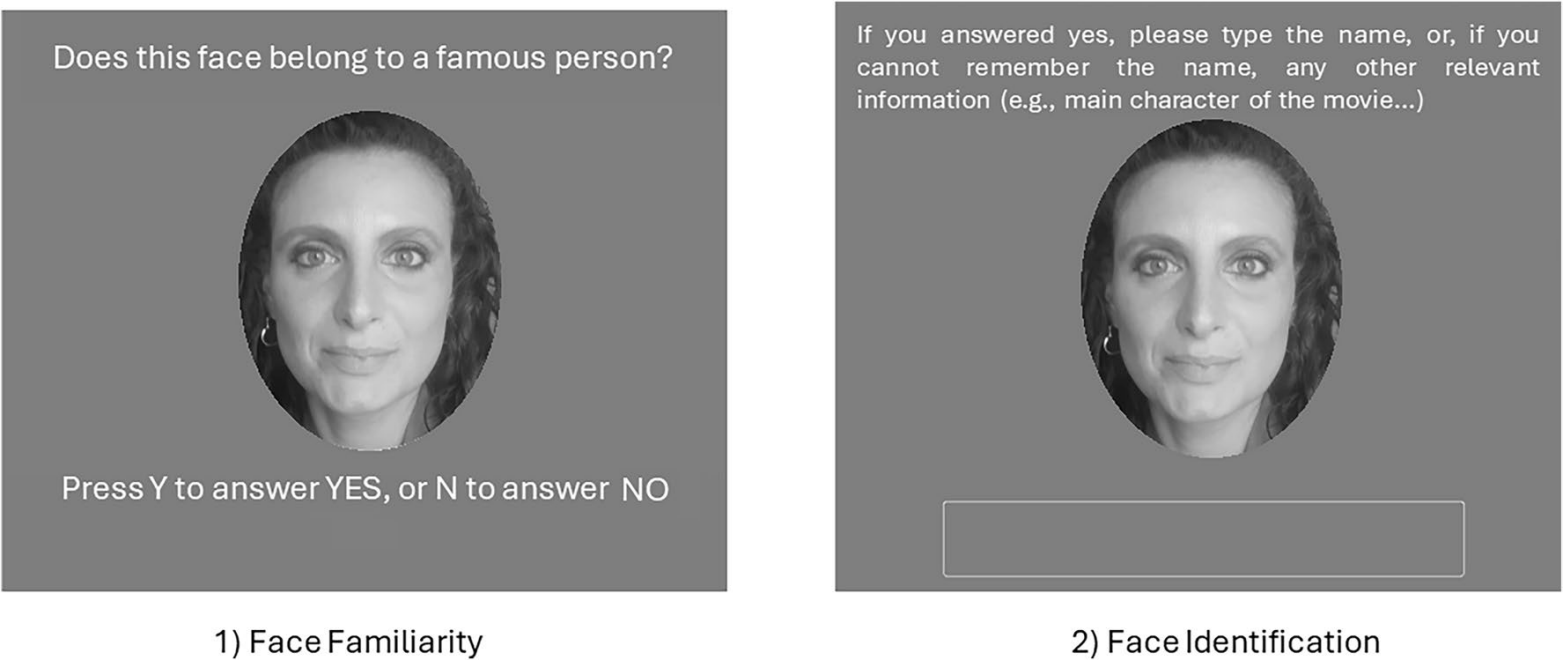
A trial example of the first phase of the IT-FFT (the second phase assessing “Name Familiarity”, is not shown here). In the first step (Face Familiarity), participants are prompted to indicate whether they recognize the presented face as famous with a binary response (Yes/No). Following this, the second (Face Identification) step focuses on identification, where participants are required to provide the name or other identifiable information corresponding to the potentially recognized face. It's important to note that (i) the original version of this test uses the Italian language; (ii) the face depicted here is not included in the actual test, serving solely for illustrative purposes; (iii) informed consent for the publication of identifying image (face) in an online open-access publication, was obtained from the individual depicted in the trial example. When participants respond to the second step, a new trial (i.e., face) is presented. At the end, the checklist for the “Name Familiarity” assessment is provided.

Participants took part in the study remotely through the online platform OpenLab^36^. The IT-FFT was administered through a computer-based interface that presented all the stimuli, one at a time, in randomized order across participants. Each stimulus (i.e., a face) was presented at the center of the screen and the participant was instructed to carefully view each photograph and complete a two-phase procedure. During the first phase (see Fig. 1), each trial presented a single face, and participants were required to dichotomously classify it as either famous or non-famous (*Face Familiarity*). If the participant indicated familiarity with the face, they were prompted to type (into a text input field) the name of the depicted individual (*Face Identification*), or any other relevant semantic information (e.g., as the title of one of their movies or songs). This initial phase comprised 100 trials (i.e., the 50 famous faces and 50 non-famous faces) presented in a randomized order to minimize sequence bias. Subsequently, participants proceeded to the second phase of the test, where they were shown a final checklist containing the names of all 50 famous individuals included in the FFT-IT and previously seen during the first phase. Participants were then asked to indicate the names of celebrities with whom they were completely unfamiliar, meaning that they could have not been able to recognize during the first phase. In this way, recognition accuracy is distinguished from the potential influence of general-culture knowledge. This final checklist was administered at the end of the task after all famous/non-famous faces were shown. The *Name familiarity score* was calculated as: “50—N of names they indicated to be unfamiliar”; this difference gives the actual number of celebrities they could be able to recognize. There were no imposed time limits in all three steps of the task.

#### Measures

Two indices were computed for statistical analysis:

- *Face identification*: One point was given for each correct celebrity identification. Identification could be made by name or other specific biographical information (e.g.,” main character of the movie…”). Name spelling errors were not considered mistakes; missing responses, incorrect answers, and responses with insufficient or overly general information (i.e., general answers such as “actor” or “singer”) were assigned a score of 0. A final index of *Face identification accuracy (in %)* was given for each participant and calculated as follows: (Face identification/Name familiarity score) * 100. This approach ensures that participants' accuracy reflects their performance relative to their self-reported knowledge of famous personalities, rather than a fixed total number of targets (see paragraph 2.2.2 Procedure).
- *Face familiarity*: Scoring for this index was based on signal detection theory^37^. Four distinct outcomes were computed: false alarms and correct rejections for non-famous faces; misses and hits for famous faces. For d-prime (*d′*) calculation “false alarms” (F) signified instances where participants incorrectly identified non-famous faces as famous, “correct rejections” indicated the accurate classification of non-famous faces as non-famous, “misses” denoted that participants failed to identify famous faces correctly; “hits” (H) indicated the successful recognition of famous faces. Increasing values of d’ refer to a greater sensitivity to a given signal^44^. Additionally, we computed response bias (*β*). An observer who is maximizing H while minimizing FA will have a β that is equal to 1.00 (i.e., no bias). A value of β below 1.00 represents a liberal tendency, i.e., to report most of the times that the target is present, while a high value of β 1 (i.e., above 1.0) represents a conservative tendency, i.e., to report most of the times that the target is absent^38^. Although signal detection measures may not be of practical use during clinical assessment, we chose to integrate *d’* to provide a thorough understanding of face recognition abilities. While traditional accuracy measures offer general insights, *d’* allows for a deeper analysis of sensitivity and response criteria, facilitating comparisons across studies^39^. Face recognition memory involves two distinct processes: recollection and familiarity. Familiarity, operating within a signal detection framework, is influenced by factors like frequency and intensity of previous exposures^40,41^. It involves acknowledging that a face is known or has been encountered before, without necessarily being able to identify it. For instance, individuals may express that a face seems familiar but struggle to assign a specific identity to it^42^. Recollection, or face identification, on the other hand, involves conscious retrieval of specific biographical details. Face identification requires accessing stored information from memory, triggered automatically by familiar faces^30,42^. This dissociation is supported by neuroimaging studies^43,44^, and it is often explored in recognition tests^45^. Signal detection theory thus, though less known in clinical settings, enhances the understanding of face recognition abilities^32,46^.

#### Prosopagnosia Index-20

All participants completed the Italian version of the Prosopagnosia Index-20 (PI-20)^47^, a self-report measure of face subjective recognition abilities. Twenty statements reflecting face recognition experiences are included in the scale; respondents indicate how accurately the statements describe them on a five-point scale (from 1 = “Totally disagree” to 5 = “Totally agree”). Scores can vary between 20 and 100. A higher score indicates more subjective problems with face recognition.

### Ethics approval and consent to participate

All procedures performed in studies involving human participants were in accordance with the ethical standards of the institutional and/or national research committee and with the 1964 Helsinki Declaration and its later amendments or comparable ethical standards. The study was approved by the Ethics Committee of the University of Bari “Aldo Moro” (protocol number: ET-19-01). Written Informed consent was obtained from all participants included in the study. Furthermore the individual depicted in the Fig. 1 gave informed consent to the publication of her image (face) in an online open-access publication.

## Results

The *M* Face identification accuracy score was 77.61% (*SD* = 14.94), the *M* d’ score for Face familiarity was 2.61 (*SD* = 0.77), and the *M* β score was 4.15 (*SD* = 4.11).

To ascertain the correlation between PI-20 and face recognition accuracy, Pearson’s correlations, their 95% confidence intervals, *p*-values, and r^2^ between the two aforementioned variables were computed for the whole sample and the sample split according to sex, age, and schooling (for age and schooling a median split has been computed). An analysis of variance (ANOVA) was conducted on Face identification accuracy as outcome and with sex, age, and schooling as categorical predictors. Finally, two ANOVAs were conducted on d’ and β scores as outcomes, respectively, and with sex, age and schooling as categorical predictors. Results indicate a statistically significant low-to-medium negative correlation (r =− 0.245, *p* < 0.001) between IT-FFT and PI-20 (95% CI from − 0.332 to − 0.156; r^2^ = 0.060). All correlations reached significance with the exclusion of males younger than 25 and for the group of males in general, for which no significant correlations were found (Table 1).

**Table 1:** Pearson’s correlations between PI-20 and face recognition accuracy percentage scores for the whole sample and for the sample split according to sex and age categories.

Sex	Age	Correlation (95% CI)	*p*-value	r^2^
Females	≤ 25	− 0.283 (− 0.411, − 0.144)	< 0.001	0.080
Females	> 25	− 0.373 (− 0.509, − 0.220)	< 0.001	0.139
Males	≤ 25	0.082 (− 0.223, 0.373)	0.599	0.007
Males	> 25	− 0.243 (− 0.450, − 0.013)	0.039	0.059
Both	≤ 25	− 0.216 (− 0.337, − 0.088)	0.001	0.047
Both	> 25	− 0.326 (− 0.442, − 0.199)	< 0.001	0.106
Females	Any	− 0.295 (− 0.392, − 0.192)	< 0.001	0.087
Males	Any	− 0.117 (− 0.294, 0.067)	0.212	0.014
Both	Any	− 0.245 (− 0.332, − 0.156)	< 0.001	0.060

A three-way ANOVA was conducted on Face identification accuracy as the outcome and with sex, age, and schooling as categorical predictors. The main effect of Age was statistically significant (F_1,429_ = 26.75, *p* < 0.001, *η*_*p*_^*2*^ = 0.06), with older participants (> 25 years old) reaching higher accuracy (*M* = 81.11%, *SD* = 14.56) than younger (≤ 25 years old) ones (*M* = 74.36%, *SD* = 14.62). No other statistically significant main effects or interactions were found.

**Figure 2:**
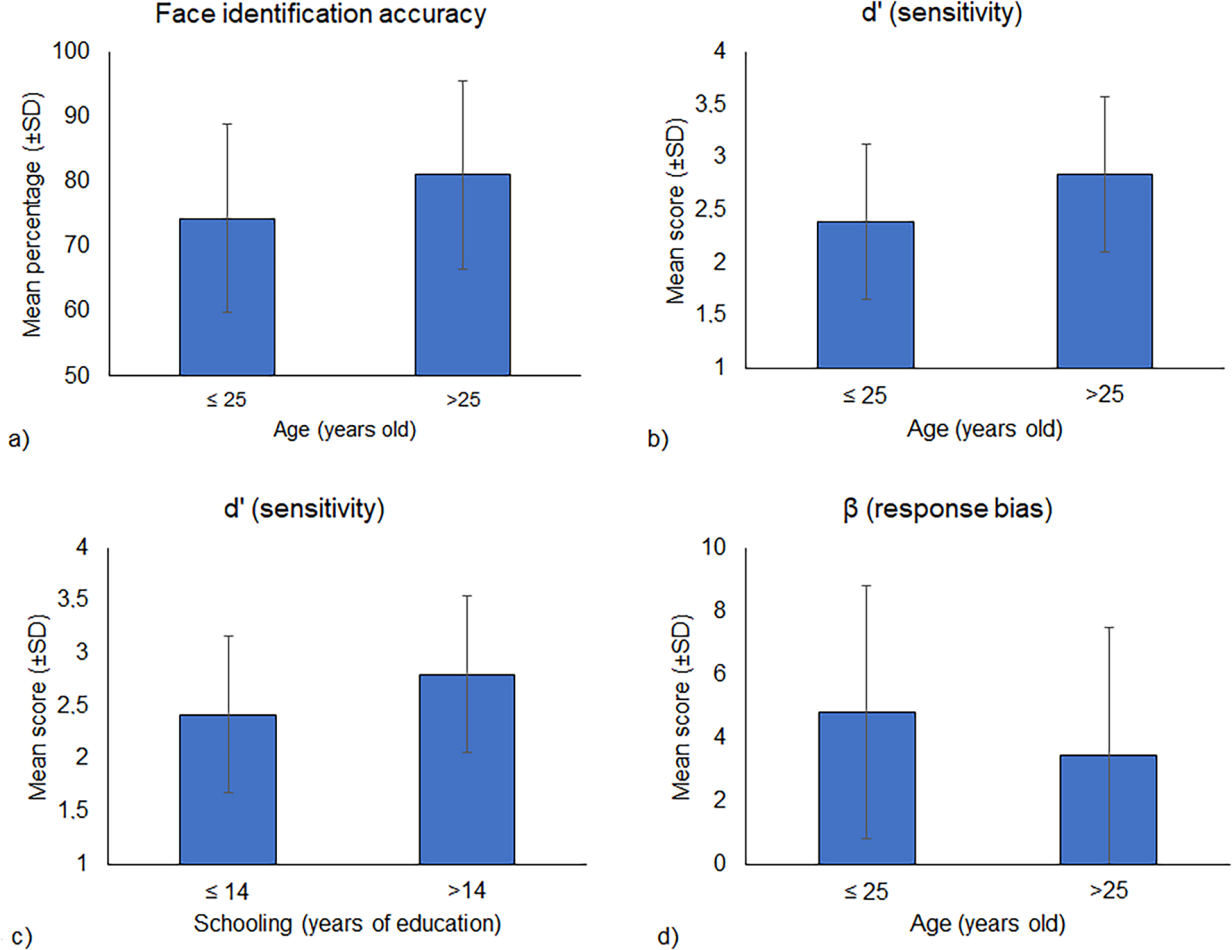
Main effect of age on accuracy (**a**) and sensitivity (**b**) scores; main effect of schooling on sensitivity score (**c**); main effect of age on response bias score (**d**).

Two three-way ANOVAs were conducted on d′ and β scores as outcomes, respectively, with sex, age, and schooling as categorical predictors. Concerning d′ scores, the main effects of Age (F_1,428_ = 43.56, *p* < 0.001, *η*_*p*_^*2*^ = 0.09) and Schooling (F_1,428_ = 8.53, *p* = 0.004, *η*_*p*_^*2*^ = 0.02) were statistically significant. Participants ≤ 25 years old obtained lower d’ scores (*M* = 2.39; *SD* = 0.70) as compared with older (> 25 years old) ones (*M* = 2.84; *SD* = 0.77). Moreover, participants with lower education (≤ 14 years) obtained lower d’ scores (*M* = 2.42; *SD* = 0.75) than those with higher (≥ 14 years) education (*M* = 2.80, *SD* = 0.74). No other main effects or interactions were found. Analysis of β scores indicated a main effect of Age (F_1,428_ = 10.99, *p* < 0.001, *η*_*p*_^*2*^ = 0.03). Participants ≤ 25 years old obtained higher β scores (*M* = 4.79, *SD* = 4.34) than older (> 25 years old) participants (*M* = 3.46; *SD* = 3.74). No other main or interaction effects were found. Figure 2 shows all the significant main effects.

Finally, we provide conversion of raw scores to T scores of face recognition accuracy for young (≤ 25 years) and adult (> 25 years) people, respectively (Table 2 and Supplementary Table 1 and 2 for further psychometric indices). Differentiated scores for the two age groups were computed to account for the influence that age had in the ANOVA model.

**Table 2:** Conversion of raw scores and Equivalent Scores (ES) of IT-FFT to T scores for people with less than or equal to 25 years of age, and for people with more than 25 years of age. Raw scores and T-Points scores were ranked on a 5-point scale in which an equivalent score of 0 is critical (below the cut-off value established as the lowest 5% of scores) and an equivalent score of 4 is beyond the median for the normative population.

ES	Age ≤ 25 years		Age > 25 years	
	Raw scores	T-Points	Raw scores	T-Points
0	≤ 46	≤ 33	≤ 49	≤ 33
1	47–63	34–41	50–70	34–41
2	64–70	42–45	71–79	42–46
3	71–76	46–50	80–85	47–50
4	≥ 77	≥ 51	≥ 86	≥ 51

## Discussion

In this study, we developed and collected normative data for a novel computer-based test, the Italian Famous Face Test (IT-FFT), on an Italian sample. Analysis of normative data revealed that adults above 25 years old exhibited greater facial recognition accuracy compared to young adults aged 18 to 25 at the IF-FFT. Interestingly, while schooling affected the d’ score (sensitivity), overall accuracy remained unaffected by sex or educational background within the sample.

Our first aim was to equip clinicians with a reliable tool designed to assess face recognition on the Italian population, providing robust normative data. Notably, face recognition deficits may appear as primary features in conditions such as congenital prosopagnosia^27^, or as a critical symptom in conditions such as autism and other neurodegenerative and neuropsychiatric conditions^21,48^. Rizzo et al. published the first normative data for an Italian famous face test in 2002^15^. Their multiple regression analyses revealed that age and education, but not sex, significantly influenced familiarity decisions and naming (i.e., identification). However, as suggested by the authors *“any test involving proper names and famous faces is the need to constantly renew the material, since people who are famous at present could well become unknown in the future”*^15^. Despite this 20-year gap and the stimuli updates, our data were largely consistent with Rizzo et al.^15^. In addition to the stimuli update, we chose to crop the images into oval shapes. This adjustment was made to remove external facial features, such as hairstyle, which could serve as a cue for identity recognition, especially in individuals with prosopagnosia^49^. Additionally, beyond evaluating face identification accuracy, we explored two supplementary indices based on signal detection theory: d' (measuring the capability to discriminate between famous and non-famous faces) and β (assessing the rate of false positives). In line with existing theories, the construct of recognition memory is commonly divided into two processes: recollection and familiarity. Although these processes are not entirely independent, familiarity is described as a continuous measure within the framework of signal detection theory (d’), influenced by factors like the quantity, intensity, and variability of past exposures^40,50^. Recollection is, instead, an attention-demanding process that leads to the conscious recollection of prior information—face, in this case (accuracy). Familiarity and recollection, therefore, are different in terms of memory content (amount of ‘context’) and underlying processes (recall or no recall), possibly making independent contributions to memory judgment^51,52^. This dual measurement approach allows for a more nuanced analysis of memory performance: studying both familiarity and recollection can help differentiate between different types of memory impairments; for instance, hippocampal pathologies are associated with spared familiarity but compromised recollection, while the contrary happens for lesion to the anterior temporal lobe with^25,53^.

The IT-FFT highlighted a clear distinction in face recognition abilities between different age groups. Older participants (≥ 25 years old) consistently showed higher face identification accuracy, as well as higher d’ and lower β scores compared to their younger counterparts (≤ 25 years old), meaning that individuals aged 25 and older demonstrated more accurate identification of faces, as well as more elevated sensitivity, indicating a heightened ability to distinguish between familiar and unfamiliar faces. In addition, older participants exhibited a more liberal approach to familiarity decisions (as indicated by a lower β score), suggesting a potential tendency on false identifications. This contrasts with younger participants, who, with higher β scores, demonstrated a greater inclination to report the target was not famous even when it showed a famous face. A potential explanation for these results is that younger individuals might be still developing these skills and have had less exposure to some famous faces in the test, leading to lower face identification accuracy and sensitivity (d’) in discriminating between famous and non-famous faces^54^. This trend is also consistent with the current literature indicating that face recognition ability tends to improve and stabilize with age^34^: older individuals likely benefit from cumulative exposure to famous faces, thus expanding their knowledge about various famous characters included in the IF-FFT. When assessing familiarity, a strong impact of education on d’ scores was evident in our data. Participants with more than 14 years of education demonstrated higher sensitivity in discriminating famous faces than those with lower education. This finding suggests that schooling influences the capacity for discriminating familiar faces; it may be possible that higher education contributes to enhanced attentional mechanisms or memory processes crucial for face recognition tasks^55,56^. Furthermore, greater education might result in a broader general knowledge involving media celebrities and historical characters knowledge included in the IT-FFT. On the other hand, the sex of our participant did not influence any index of famous face identification, familiarity, and false alarm rate, confirming a substantial comparable ability in face recognition between men and females^15^.

Moreover, based on the accuracy results, we generated the normative table (Table 2) by converting raw scores into *t*-points and then into equivalent scores for the two distinct age groups (≤ 25 and > 25 years old). It's important to note that the interpretation of T scores should be considered in a clinical context. An isolated T score may not provide a complete picture, and additional factors, such as the individual's history, context, and the specific assessment being used, should be considered.

## Conclusions

In this study, we provided normative data for the IT-FFT, a novel test designed to detect face recognition issues in the Italian population. Through our data analysis, age emerged as the key determinant influencing IT-FFT scores (over both sex and schooling). Thus, contrary to the previous famous face test^15^, we did not report a correction grid separated by years and schooling, rather we developed separate equivalent scores for the 2 distinct age groups (i.e., over and under 25 years old). This approach was pursued since our sample distribution was not homogenous by age groups or years of school, despite being much larger than those collected two decades ago. A limitation of the IT-FFT is that, while this test can provide insights into the ability to recognize well-known faces, assessing identification skills and familiarity, our data analysis strategy may not fully capture the comprehensive aspects of face recognition abilities, as other tasks related to face recognition were not assessed. This limitation highlights the need for future studies to include measures of reliability and validity to ensure robust and meaningful conclusions. Administration of this test, together with others such as the CFPT and the CFMT^12,13^ can provide a comprehensive insight into individual face identification dimensions (i.e., face perception, face memory, and face recognition). Additionally, it’s important to acknowledge the limitations inherent in self-report measures for assessing face recognition abilities; thus, our study does not assert the PI-20 as a standalone diagnostic instrument. Instead, we aimed to elucidate its potential role alongside objective performance measures, shedding light on the relationship between self-reported abilities and actual performance. Despite the valuable insights self-report measures may offer, particularly in screening contexts, the inherent limitations necessitate a cautious interpretation of findings.

In conclusion, the development of a reliable and complete battery for the evaluation of face recognition deficits is crucial, particularly in facilitating early diagnosis across a spectrum of conditions. Disorders such as Alzheimer's disease, frontotemporal dementia, ASD, and others often manifest with difficulties in facial recognition. In some cases, face perception/memory is the sole symptom such as in prosopagnosia that can be labeled as apperceptive, when patients are also unable to match unfamiliar faces, or as associative, when deficit resulting from a disconnection between the face analysis and the face storage levels. A comprehensive test battery should effectively distinguish between these conditions, enabling not just prompt diagnoses and interventions but also fostering a more profound comprehension of their courses. This paper introduces clinicians to a novel fast and easy-to-use tool— the IT-FFT— designed to assess Italian famous face identification, furthering the pursuit of these objectives.

### Supplementary Information

Supplementary Tables.

## Data Availability

All the data is provided within the manuscript or supplementary materials file. Other data/test information can be found here: 10.6084/m9.figshare.25603887.
