# Supplementary material for: Normative data of the Italian Famous Face Test
Source: Sci Rep. 2024 Jul 3;14:15276. doi: 10.1038/s41598-024-66252-1 (PMC11222389; doi:10.1038/s41598-024-66252-1)
Supplement: Supplementary file 1 — Supplementary Tables. [file 41598_2024_66252_MOESM1_ESM.docx]

| ID | Sex | Age | Age_ binary | School | School_binary | PI-20 | Recognized | Not Known (checklist) | Known (checklist) | Normalized accuracy | Fam_HIT | Fam_MISS | Fam_FA | Fam_CR |
| --- | --- | --- | --- | --- | --- | --- | --- | --- | --- | --- | --- | --- | --- | --- |
| S067 | F | 27 | >25 | 16 | >15 | 63 | 11 | 9 | 41 | 26,83 | 17 | 33 | 0 | 50 |
| S204 | F | 42 | >25 | 18 | >15 | 57 | 16 | 1 | 49 | 32,65 | 17 | 33 | 1 | 49 |
| S320 | F | 19 | <=25 | 8 | <=14 | 41 | 18 | 0 | 50 | 36 | 31 | 19 | 11 | 39 |
| S399 | M | 23 | <=25 | 17 | >15 | 31 | 12 | 17 | 33 | 36,36 | 27 | 23 | 1 | 49 |
| S303 | M | 28 | >25 | 18 | >15 | 42 | 18 | 2 | 48 | 37,5 | 20 | 30 | 1 | 49 |
| S412 | M | 60 | >25 | 13 | <=14 | 41 | 19 | 2 | 48 | 39,58 | 36 | 14 | 10 | 40 |
| S129 | M | 19 | <=25 | 13 | <=14 | 33 | 15 | 13 | 37 | 40,54 | 18 | 32 | 7 | 43 |
| S429 | M | 26 | >25 | 16 | >15 | 36 | 13 | 18 | 32 | 40,63 | 18 | 32 | 0 | 50 |
| S260 | M | 19 | <=25 | 13 | <=14 | 32 | 18 | 6 | 44 | 40,91 | 32 | 18 | 13 | 37 |
| S189 | F | 19 | <=25 | 13 | <=14 | 55 | 14 | 16 | 34 | 41,18 | 19 | 31 | 6 | 44 |
| S229 | F | 55 | >25 | 13 | <=14 | 59 | 19 | 4 | 46 | 41,3 | 24 | 26 | 1 | 49 |
| S142 | M | 22 | <=25 | 13 | <=14 | 54 | 11 | 24 | 26 | 42,31 | 16 | 34 | 3 | 47 |
| S209 | M | 22 | <=25 | 16 | >15 | 41 | 21 | 1 | 49 | 42,86 | 23 | 27 | 4 | 46 |
| S405 | F | 58 | >25 | 15 | >15 | 63 | 21 | 2 | 48 | 43,75 | 46 | 4 | 27 | 23 |
| S344 | F | 19 | <=25 | 13 | <=14 | 45 | 18 | 9 | 41 | 43,9 | 39 | 11 | 2 | 48 |
| S302 | F | 19 | <=25 | 13 | <=14 | 45 | 20 | 6 | 44 | 45,45 | 35 | 15 | 11 | 39 |
| S255 | F | 19 | <=25 | 13 | <=14 | 64 | 15 | 17 | 33 | 45,45 | 16 | 34 | 4 | 46 |
| S293 | M | 19 | <=25 | 14 | <=14 | 36 | 20 | 6 | 44 | 45,45 | 25 | 25 | 1 | 49 |
| S350 | F | 20 | <=25 | 13 | <=14 | 45 | 16 | 15 | 35 | 45,71 | 17 | 33 | 0 | 50 |
| S213 | F | 19 | <=25 | 14 | <=14 | 61 | 16 | 15 | 35 | 45,71 | 19 | 31 | 1 | 49 |
| S230 | F | 20 | <=25 | 14 | <=14 | 47 | 19 | 9 | 41 | 46,34 | 35 | 15 | 0 | 50 |
| S296 | F | 20 | <=25 | 13 | <=14 | 35 | 20 | 7 | 43 | 46,51 | 30 | 20 | 1 | 49 |
| S058 | M | 31 | >25 | 18 | >15 | 30 | 21 | 5 | 45 | 46,67 | 43 | 7 | 2 | 48 |
| S264 | F | 33 | >25 | 20 | >15 | 31 | 21 | 5 | 45 | 46,67 | 43 | 7 | 4 | 46 |
| S331 | M | 22 | <=25 | 17 | >15 | 30 | 20 | 8 | 42 | 47,62 | 22 | 28 | 3 | 47 |
| S212 | M | 22 | <=25 | 16 | >15 | 36 | 21 | 6 | 44 | 47,73 | 41 | 9 | 24 | 26 |
| S163 | F | 25 | <=25 | 18 | >15 | 58 | 22 | 4 | 46 | 47,83 | 26 | 24 | 2 | 48 |
| S194 | F | 19 | <=25 | 13 | <=14 | 69 | 13 | 23 | 27 | 48,15 | 23 | 27 | 10 | 40 |
| S138 | M | 26 | >25 | 17 | >15 | 83 | 22 | 5 | 45 | 48,89 | 33 | 17 | 3 | 47 |
| S283 | F | 19 | <=25 | 13 | <=14 | 30 | 23 | 3 | 47 | 48,94 | 32 | 18 | 8 | 42 |
| S418 | F | 51 | >25 | 16 | >15 | 47 | 23 | 3 | 47 | 48,94 | 48 | 2 | 10 | 40 |
| S356 | M | 54 | >25 | 8 | <=14 | 42 | 24 | 2 | 48 | 50 | 43 | 7 | 16 | 34 |
| S248 | M | 19 | <=25 | 13 | <=14 | 31 | 23 | 4 | 46 | 50 | 28 | 22 | 4 | 46 |
| S222 | F | 19 | <=25 | 13 | <=14 | 36 | 17 | 16 | 34 | 50 | 23 | 27 | 0 | 50 |
| S026 | F | 30 | >25 | 18 | >15 | 77 | 24 | 4 | 46 | 52,17 | 34 | 16 | 0 | 50 |
| S126 | M | 42 | >25 | 13 | <=14 | 39 | 26 | 1 | 49 | 53,06 | 29 | 21 | 3 | 47 |
| S401 | M | 54 | >25 | 8 | <=14 | 37 | 24 | 5 | 45 | 53,33 | 33 | 17 | 3 | 47 |
| S256 | M | 19 | <=25 | 13 | <=14 | 42 | 23 | 7 | 43 | 53,49 | 28 | 22 | 1 | 49 |
| S226 | F | 56 | >25 | 13 | <=14 | 24 | 27 | 1 | 49 | 55,1 | 38 | 12 | 2 | 48 |
| S414 | F | 20 | <=25 | 13 | <=14 | 30 | 21 | 12 | 38 | 55,26 | 32 | 18 | 3 | 47 |
| S064 | F | 19 | <=25 | 13 | <=14 | 35 | 26 | 3 | 47 | 55,32 | 30 | 20 | 6 | 44 |
| S246 | M | 22 | <=25 | 16 | >15 | 27 | 24 | 7 | 43 | 55,81 | 28 | 22 | 0 | 50 |
| S031 | M | 22 | <=25 | 16 | >15 | 54 | 28 | 0 | 50 | 56 | 32 | 18 | 1 | 49 |
| S134 | F | 24 | <=25 | 18 | >15 | 41 | 27 | 2 | 48 | 56,25 | 38 | 12 | 0 | 50 |
| S251 | F | 19 | <=25 | 13 | <=14 | 46 | 22 | 11 | 39 | 56,41 | 37 | 13 | 4 | 46 |
| S090 | F | 20 | <=25 | 14 | <=14 | 46 | 26 | 4 | 46 | 56,52 | 36 | 14 | 7 | 43 |
| S381 | F | 22 | <=25 | 16 | >15 | 55 | 26 | 4 | 46 | 56,52 | 33 | 17 | 1 | 49 |
| S258 | F | 19 | <=25 | 14 | <=14 | 36 | 25 | 6 | 44 | 56,82 | 38 | 12 | 13 | 37 |
| S262 | F | 21 | <=25 | 16 | >15 | 33 | 20 | 15 | 35 | 57,14 | 22 | 28 | 2 | 48 |
| S250 | F | 20 | <=25 | 13 | <=14 | 39 | 15 | 24 | 26 | 57,69 | 19 | 31 | 0 | 50 |
| S267 | F | 19 | <=25 | 13 | <=14 | 55 | 26 | 5 | 45 | 57,78 | 33 | 17 | 7 | 43 |
| S020 | F | 31 | >25 | 18 | >15 | 73 | 26 | 5 | 45 | 57,78 | 29 | 21 | 0 | 50 |
| S365 | F | 57 | >25 | 8 | <=14 | 28 | 22 | 12 | 38 | 57,89 | 33 | 17 | 3 | 47 |
| S314 | F | 23 | <=25 | 17 | >15 | 29 | 27 | 4 | 46 | 58,7 | 37 | 13 | 3 | 47 |
| S195 | F | 20 | <=25 | 8 | <=14 | 44 | 20 | 16 | 34 | 58,82 | 33 | 17 | 2 | 48 |
| S346 | F | 21 | <=25 | 14 | <=14 | 48 | 25 | 8 | 42 | 59,52 | 40 | 10 | 1 | 49 |
| S029 | M | 35 | >25 | 13 | <=14 | 37 | 30 | 0 | 50 | 60 | 37 | 13 | 7 | 43 |
| S359 | F | 20 | <=25 | 14 | <=14 | 49 | 23 | 12 | 38 | 60,53 | 24 | 26 | 1 | 49 |
| S168 | M | 35 | >25 | 21 | >15 | 53 | 28 | 4 | 46 | 60,87 | 29 | 21 | 0 | 50 |
| S218 | F | 20 | <=25 | 15 | >15 | 50 | 27 | 6 | 44 | 61,36 | 33 | 17 | 2 | 48 |
| S038 | M | 35 | >25 | 16 | >15 | 59 | 31 | 0 | 50 | 62 | 41 | 9 | 16 | 34 |
| S135 | M | 19 | <=25 | 13 | <=14 | 54 | 23 | 13 | 37 | 62,16 | 30 | 20 | 1 | 49 |
| S306 | F | 19 | <=25 | 13 | <=14 | 32 | 20 | 18 | 32 | 62,5 | 22 | 28 | 0 | 50 |
| S100 | F | 19 | <=25 | 13 | <=14 | 37 | 25 | 10 | 40 | 62,5 | 27 | 23 | 1 | 49 |
| S234 | F | 19 | <=25 | 13 | <=14 | 40 | 25 | 10 | 40 | 62,5 | 27 | 23 | 2 | 48 |
| S095 | F | 24 | <=25 | 18 | >15 | 33 | 27 | 7 | 43 | 62,79 | 33 | 17 | 1 | 49 |
| S318 | F | 22 | <=25 | 17 | >15 | 39 | 22 | 15 | 35 | 62,86 | 28 | 22 | 0 | 50 |
| S382 | F | 19 | <=25 | 22 | >15 | 51 | 22 | 15 | 35 | 62,86 | 34 | 16 | 1 | 49 |
| S108 | F | 27 | >25 | 15 | >15 | 70 | 29 | 4 | 46 | 63,04 | 31 | 19 | 3 | 47 |
| S183 | F | 18 | <=25 | 13 | <=14 | 45 | 12 | 31 | 19 | 63,16 | 15 | 35 | 3 | 47 |
| S186 | F | 19 | <=25 | 13 | <=14 | 40 | 24 | 12 | 38 | 63,16 | 37 | 13 | 1 | 49 |
| S317 | F | 20 | <=25 | 14 | <=14 | 56 | 31 | 1 | 49 | 63,27 | 38 | 12 | 4 | 46 |
| S328 | F | 21 | <=25 | 16 | >15 | 42 | 31 | 1 | 49 | 63,27 | 40 | 10 | 3 | 47 |
| S240 | F | 46 | >25 | 8 | <=14 | 76 | 26 | 9 | 41 | 63,41 | 39 | 11 | 1 | 49 |
| S004 | M | 27 | >25 | 16 | >15 | 40 | 30 | 3 | 47 | 63,83 | 36 | 14 | 1 | 49 |
| S295 | F | 19 | <=25 | 13 | <=14 | 49 | 25 | 11 | 39 | 64,1 | 33 | 17 | 7 | 43 |
| S199 | F | 19 | <=25 | 13 | <=14 | 41 | 29 | 5 | 45 | 64,44 | 32 | 18 | 1 | 49 |
| S068 | M | 19 | <=25 | 13 | <=14 | 39 | 28 | 7 | 43 | 65,12 | 30 | 20 | 1 | 49 |
| S301 | F | 22 | <=25 | 16 | >15 | 30 | 30 | 4 | 46 | 65,22 | 35 | 15 | 0 | 50 |
| S276 | F | 21 | <=25 | 13 | <=14 | 39 | 32 | 1 | 49 | 65,31 | 35 | 15 | 1 | 49 |
| S021 | F | 29 | >25 | 18 | >15 | 37 | 32 | 1 | 49 | 65,31 | 45 | 5 | 19 | 31 |
| S377 | F | 19 | <=25 | 13 | <=14 | 46 | 23 | 15 | 35 | 65,71 | 35 | 15 | 7 | 43 |
| S268 | F | 19 | <=25 | 14 | <=14 | 34 | 29 | 6 | 44 | 65,91 | 34 | 16 | 4 | 46 |
| S409 | F | 48 | >25 | 14 | <=14 | 48 | 33 | 0 | 50 | 66 | 43 | 7 | 7 | 43 |
| S039 | F | 26 | >25 | 18 | >15 | 56 | 33 | 0 | 50 | 66 | 35 | 15 | 2 | 48 |
| S347 | F | 54 | >25 | 8 | <=14 | 56 | 32 | 2 | 48 | 66,67 | 38 | 12 | 4 | 46 |
| S348 | F | 57 | >25 | 10 | <=14 | 45 | 32 | 2 | 48 | 66,67 | 47 | 3 | 12 | 38 |
| S224 | F | 19 | <=25 | 13 | <=14 | 39 | 32 | 2 | 48 | 66,67 | 38 | 12 | 2 | 48 |
| S241 | F | 19 | <=25 | 13 | <=14 | 43 | 22 | 17 | 33 | 66,67 | 35 | 15 | 4 | 46 |
| S219 | F | 20 | <=25 | 13 | <=14 | 27 | 30 | 5 | 45 | 66,67 | 42 | 8 | 9 | 41 |
| S432 | M | 35 | >25 | 13 | <=14 | 34 | 20 | 20 | 30 | 66,67 | 28 | 22 | 5 | 45 |
| S361 | F | 22 | <=25 | 16 | >15 | 34 | 22 | 17 | 33 | 66,67 | 31 | 19 | 0 | 50 |
| S334 | M | 24 | <=25 | 17 | >15 | 39 | 18 | 23 | 27 | 66,67 | 34 | 16 | 5 | 45 |
| S411 | M | 24 | <=25 | 18 | >15 | 33 | 33 | 1 | 49 | 67,35 | 46 | 4 | 9 | 41 |
| S158 | M | 27 | >25 | 18 | >15 | 36 | 29 | 7 | 43 | 67,44 | 32 | 18 | 0 | 50 |
| S391 | F | 55 | >25 | 13 | <=14 | 56 | 27 | 10 | 40 | 67,5 | 29 | 21 | 0 | 50 |
| S015 | F | 28 | >25 | 18 | >15 | 41 | 34 | 0 | 50 | 68 | 37 | 13 | 1 | 49 |
| S337 | F | 20 | <=25 | 8 | <=14 | 40 | 32 | 3 | 47 | 68,09 | 38 | 12 | 0 | 50 |
| S338 | F | 20 | <=25 | 8 | <=14 | 36 | 30 | 6 | 44 | 68,18 | 36 | 14 | 4 | 46 |
| S181 | F | 19 | <=25 | 13 | <=14 | 41 | 26 | 12 | 38 | 68,42 | 30 | 20 | 4 | 46 |
| S279 | F | 19 | <=25 | 13 | <=14 | 47 | 26 | 12 | 38 | 68,42 | 40 | 10 | 16 | 34 |
| S282 | F | 19 | <=25 | 14 | <=14 | 54 | 26 | 12 | 38 | 68,42 | 30 | 20 | 2 | 48 |
| S088 | F | 23 | <=25 | 13 | <=14 | 28 | 22 | 18 | 32 | 68,75 | 34 | 16 | 3 | 47 |
| S060 | F | 31 | >25 | 18 | >15 | 56 | 31 | 5 | 45 | 68,89 | 43 | 7 | 15 | 35 |
| S271 | F | 21 | <=25 | 16 | >15 | 40 | 29 | 8 | 42 | 69,05 | 33 | 17 | 3 | 47 |
| S103 | F | 26 | >25 | 21 | >15 | 31 | 29 | 8 | 42 | 69,05 | 31 | 19 | 0 | 50 |
| S353 | M | 20 | <=25 | 13 | <=14 | 52 | 27 | 11 | 39 | 69,23 | 30 | 20 | 0 | 50 |
| S435 | M | 27 | >25 | 18 | >15 | 47 | 34 | 1 | 49 | 69,39 | 37 | 13 | 12 | 38 |
| S059 | F | 32 | >25 | 18 | >15 | 33 | 34 | 1 | 49 | 69,39 | 42 | 8 | 2 | 48 |
| S072 | F | 30 | >25 | 13 | <=14 | 33 | 32 | 4 | 46 | 69,57 | 36 | 14 | 9 | 41 |
| S220 | M | 23 | <=25 | 16 | >15 | 44 | 23 | 17 | 33 | 69,7 | 27 | 23 | 0 | 50 |
| S397 | F | 26 | >25 | 18 | >15 | 39 | 30 | 7 | 43 | 69,77 | 39 | 11 | 1 | 49 |
| S139 | F | 43 | >25 | 18 | >15 | 28 | 30 | 7 | 43 | 69,77 | 35 | 15 | 5 | 45 |
| S049 | F | 25 | <=25 | 13 | <=14 | 58 | 35 | 0 | 50 | 70 | 36 | 14 | 0 | 50 |
| S089 | F | 20 | <=25 | 15 | >15 | 34 | 28 | 10 | 40 | 70 | 41 | 9 | 4 | 46 |
| S071 | F | 33 | >25 | 16 | >15 | 33 | 35 | 0 | 50 | 70 | 38 | 12 | 1 | 49 |
| S383 | M | 19 | <=25 | 13 | <=14 | 31 | 33 | 3 | 47 | 70,21 | 48 | 2 | 2 | 48 |
| S197 | F | 19 | <=25 | 13 | <=14 | 37 | 33 | 3 | 47 | 70,21 | 40 | 10 | 0 | 50 |
| S023 | F | 38 | >25 | 18 | >15 | 66 | 33 | 3 | 47 | 70,21 | 40 | 10 | 1 | 49 |
| S427 | M | 25 | <=25 | 17 | >15 | 36 | 19 | 23 | 27 | 70,37 | 24 | 26 | 1 | 49 |
| S311 | F | 19 | <=25 | 13 | <=14 | 29 | 31 | 6 | 44 | 70,45 | 34 | 16 | 2 | 48 |
| S203 | F | 19 | <=25 | 13 | <=14 | 42 | 31 | 6 | 44 | 70,45 | 50 |  | 7 | 43 |
| S327 | F | 20 | <=25 | 13 | <=14 | 42 | 31 | 6 | 44 | 70,45 | 37 | 13 | 1 | 49 |
| S305 | F | 19 | <=25 | 14 | <=14 | 33 | 29 | 9 | 41 | 70,73 | 35 | 15 | 4 | 46 |
| S261 | F | 22 | <=25 | 16 | >15 | 26 | 29 | 9 | 41 | 70,73 | 35 | 15 | 4 | 46 |
| S104 | F | 31 | >25 | 18 | >15 | 31 | 34 | 2 | 48 | 70,83 | 38 | 12 | 0 | 50 |
| S379 | F | 23 | <=25 | 17 | >15 | 26 | 22 | 19 | 31 | 70,97 | 35 | 15 | 6 | 44 |
| S107 | F | 53 | >25 | 18 | >15 | 44 | 32 | 5 | 45 | 71,11 | 39 | 11 | 5 | 45 |
| S259 | M | 50 | >25 | 14 | <=14 | 43 | 35 | 1 | 49 | 71,43 | 46 | 4 | 13 | 37 |
| S172 | F | 43 | >25 | 13 | <=14 | 57 | 33 | 4 | 46 | 71,74 | 43 | 7 | 0 | 50 |
| S115 | F | 21 | <=25 | 18 | >15 | 31 | 33 | 4 | 46 | 71,74 | 36 | 14 | 0 | 50 |
| S037 | M | 22 | <=25 | 13 | <=14 | 32 | 28 | 11 | 39 | 71,79 | 30 | 20 | 4 | 46 |
| S413 | F | 54 | >25 | 18 | >15 | 45 | 23 | 18 | 32 | 71,88 | 23 | 27 | 1 | 49 |
| S056 | F | 42 | >25 | 18 | >15 | 41 | 36 | 0 | 50 | 72 | 37 | 13 | 0 | 50 |
| S371 | F | 22 | <=25 | 13 | <=14 | 65 | 31 | 7 | 43 | 72,09 | 37 | 13 | 1 | 49 |
| S236 | M | 19 | <=25 | 14 | <=14 | 41 | 31 | 7 | 43 | 72,09 | 33 | 17 | 0 | 50 |
| S316 | F | 22 | <=25 | 16 | >15 | 30 | 31 | 7 | 43 | 72,09 | 35 | 15 | 2 | 48 |
| S201 | F | 19 | <=25 | 13 | <=14 | 32 | 26 | 14 | 36 | 72,22 | 32 | 18 | 2 | 48 |
| S187 | F | 20 | <=25 | 13 | <=14 | 37 | 26 | 14 | 36 | 72,22 | 28 | 22 | 0 | 50 |
| S394 | F | 19 | <=25 | 13 | <=14 | 33 | 21 | 21 | 29 | 72,41 | 23 | 27 | 5 | 45 |
| S285 | F | 20 | <=25 | 14 | <=14 | 31 | 29 | 10 | 40 | 72,5 | 29 | 21 | 0 | 50 |
| S227 | F | 23 | <=25 | 15 | >15 | 47 | 29 | 10 | 40 | 72,5 | 29 | 21 | 2 | 48 |
| S324 | M | 58 | >25 | 8 | <=14 | 67 | 32 | 6 | 44 | 72,73 | 41 | 9 | 4 | 46 |
| S265 | F | 20 | <=25 | 13 | <=14 | 52 | 32 | 6 | 44 | 72,73 | 35 | 15 | 2 | 48 |
| S358 | F | 19 | <=25 | 14 | <=14 | 32 | 24 | 17 | 33 | 72,73 | 24 | 26 | 1 | 49 |
| S025 | F | 41 | >25 | 21 | >15 | 33 | 32 | 6 | 44 | 72,73 | 40 | 10 | 0 | 50 |
| S176 | F | 27 | >25 | 19 | >15 | 38 | 35 | 2 | 48 | 72,92 | 40 | 10 | 0 | 50 |
| S349 | F | 19 | <=25 | 13 | <=14 | 39 | 22 | 20 | 30 | 73,33 | 30 | 20 | 5 | 45 |
| S110 | F | 29 | >25 | 18 | >15 | 64 | 33 | 5 | 45 | 73,33 | 34 | 16 | 2 | 48 |
| S326 | F | 46 | >25 | 13 | <=14 | 25 | 36 | 1 | 49 | 73,47 | 50 | 0 | 15 | 35 |
| S146 | F | 27 | >25 | 21 | >15 | 28 | 36 | 1 | 49 | 73,47 | 41 | 9 | 8 | 42 |
| S085 | M | 31 | >25 | 21 | >15 | 49 | 36 | 1 | 49 | 73,47 | 44 | 6 | 1 | 49 |
| S211 | F | 20 | <=25 | 13 | <=14 | 29 | 25 | 16 | 34 | 73,53 | 32 | 18 | 4 | 46 |
| S207 | F | 19 | <=25 | 13 | <=14 | 37 | 28 | 12 | 38 | 73,68 | 38 | 12 | 1 | 49 |
| S238 | F | 20 | <=25 | 13 | <=14 | 30 | 31 | 8 | 42 | 73,81 | 32 | 18 | 1 | 49 |
| S393 | F | 20 | <=25 | 14 | <=14 | 37 | 31 | 8 | 42 | 73,81 | 34 | 16 | 3 | 47 |
| S299 | F | 19 | <=25 | 13 | <=14 | 61 | 34 | 4 | 46 | 73,91 | 47 | 3 | 6 | 44 |
| S214 | M | 20 | <=25 | 13 | <=14 | 51 | 34 | 4 | 46 | 73,91 | 41 | 9 | 4 | 46 |
| S252 | F | 25 | <=25 | 17 | >15 | 35 | 34 | 4 | 46 | 73,91 | 43 | 7 | 8 | 42 |
| S430 | M | 34 | >25 | 18 | >15 | 35 | 34 | 4 | 46 | 73,91 | 34 | 16 | 0 | 50 |
| S143 | F | 31 | >25 | 21 | >15 | 42 | 34 | 4 | 46 | 73,91 | 43 | 7 | 0 | 50 |
| S367 | M | 25 | <=25 | 14 | <=14 | 44 | 37 | 0 | 50 | 74 | 44 | 6 | 8 | 42 |
| S001 | M | 26 | >25 | 18 | >15 | 28 | 37 | 0 | 50 | 74 | 44 | 6 | 2 | 48 |
| S106 | F | 60 | >25 | 8 | <=14 | 40 | 32 | 7 | 43 | 74,42 | 46 | 4 | 30 | 20 |
| S005 | F | 27 | >25 | 18 | >15 | 33 | 32 | 7 | 43 | 74,42 | 46 | 4 | 4 | 46 |
| S208 | M | 19 | <=25 | 13 | <=14 | 36 | 33 | 6 | 44 | 75 | 38 | 12 | 1 | 49 |
| S428 | M | 51 | >25 | 13 | <=14 | 49 | 30 | 10 | 40 | 75 | 33 | 17 | 2 | 48 |
| S150 | M | 35 | >25 | 21 | >15 | 32 | 36 | 2 | 48 | 75 | 42 | 8 | 0 | 50 |
| S291 | F | 18 | <=25 | 13 | <=14 | 44 | 37 | 1 | 49 | 75,51 | 41 | 9 | 0 | 50 |
| S243 | M | 20 | <=25 | 13 | <=14 | 30 | 37 | 1 | 49 | 75,51 | 38 | 12 | 1 | 49 |
| S184 | F | 20 | <=25 | 13 | <=14 | 43 | 37 | 1 | 49 | 75,51 | 41 | 9 | 2 | 48 |
| S310 | F | 19 | <=25 | 8 | <=14 | 44 | 34 | 5 | 45 | 75,56 | 45 | 5 | 0 | 50 |
| S309 | F | 19 | <=25 | 13 | <=14 | 32 | 34 | 5 | 45 | 75,56 | 43 | 7 | 5 | 45 |
| S403 | F | 22 | <=25 | 16 | >15 | 26 | 34 | 5 | 45 | 75,56 | 38 | 12 | 1 | 49 |
| S160 | F | 44 | >25 | 21 | >15 | 37 | 34 | 5 | 45 | 75,56 | 45 | 5 | 12 | 38 |
| S263 | F | 22 | <=25 | 16 | >15 | 31 | 28 | 13 | 37 | 75,68 | 33 | 17 | 0 | 50 |
| S144 | M | 35 | >25 | 13 | <=14 | 52 | 38 | 0 | 50 | 76 | 43 | 7 | 4 | 46 |
| S009 | F | 27 | >25 | 18 | >15 | 24 | 35 | 4 | 46 | 76,09 | 44 | 6 | 0 | 50 |
| S069 | F | 26 | >25 | 13 | <=14 | 47 | 32 | 8 | 42 | 76,19 | 40 | 10 | 6 | 44 |
| S116 | M | 26 | >25 | 18 | >15 | 38 | 32 | 8 | 42 | 76,19 | 36 | 14 | 2 | 48 |
| S171 | M | 24 | <=25 | 18 | >15 | 34 | 29 | 12 | 38 | 76,32 | 42 | 8 | 0 | 50 |
| S355 | F | 19 | <=25 | 8 | <=14 | 40 | 26 | 16 | 34 | 76,47 | 42 | 8 | 0 | 50 |
| S041 | M | 30 | >25 | 18 | >15 | 40 | 36 | 3 | 47 | 76,6 | 44 | 6 | 9 | 41 |
| S063 | F | 40 | >25 | 21 | >15 | 69 | 36 | 3 | 47 | 76,6 | 35 | 15 | 1 | 49 |
| S200 | F | 20 | <=25 | 14 | <=14 | 32 | 23 | 20 | 30 | 76,67 | 32 | 18 | 0 | 50 |
| S352 | F | 19 | <=25 | 13 | <=14 | 38 | 33 | 7 | 43 | 76,74 | 40 | 10 | 1 | 49 |
| S141 | F | 28 | >25 | 18 | >15 | 37 | 30 | 11 | 39 | 76,92 | 38 | 12 | 4 | 46 |
| S157 | M | 34 | >25 | 16 | >15 | 47 | 37 | 2 | 48 | 77,08 | 42 | 8 | 4 | 46 |
| S335 | F | 20 | <=25 | 13 | <=14 | 37 | 34 | 6 | 44 | 77,27 | 36 | 14 | 3 | 47 |
| S300 | F | 19 | <=25 | 8 | <=14 | 36 | 31 | 10 | 40 | 77,5 | 33 | 17 | 2 | 48 |
| S372 | F | 19 | <=25 | 13 | <=14 | 27 | 38 | 1 | 49 | 77,55 | 46 | 4 | 13 | 37 |
| S247 | F | 19 | <=25 | 13 | <=14 | 28 | 38 | 1 | 49 | 77,55 | 39 | 11 | 0 | 50 |
| S033 | M | 42 | >25 | 21 | >15 | 35 | 38 | 1 | 49 | 77,55 | 39 | 11 | 3 | 47 |
| S154 | F | 19 | <=25 | 13 | <=14 | 24 | 35 | 5 | 45 | 77,78 | 35 | 15 | 0 | 50 |
| S433 | M | 21 | <=25 | 16 | >15 | 49 | 28 | 14 | 36 | 77,78 | 31 | 19 | 1 | 49 |
| S375 | F | 23 | <=25 | 16 | >15 | 35 | 32 | 9 | 41 | 78,05 | 35 | 15 | 0 | 50 |
| S215 | F | 19 | <=25 | 13 | <=14 | 26 | 36 | 4 | 46 | 78,26 | 36 | 14 | 0 | 50 |
| S223 | F | 19 | <=25 | 13 | <=14 | 34 | 36 | 4 | 46 | 78,26 | 43 | 7 | 4 | 46 |
| S376 | F | 19 | <=25 | 13 | <=14 | 34 | 29 | 13 | 37 | 78,38 | 31 | 19 | 1 | 49 |
| S275 | F | 19 | <=25 | 13 | <=14 | 44 | 29 | 13 | 37 | 78,38 | 32 | 18 | 0 | 50 |
| S165 | F | 29 | >25 | 16 | >15 | 35 | 29 | 13 | 37 | 78,38 | 33 | 17 | 4 | 46 |
| S363 | F | 24 | <=25 | 16 | >15 | 38 | 33 | 8 | 42 | 78,57 | 41 | 9 | 2 | 48 |
| S131 | F | 21 | <=25 | 12 | <=14 | 27 | 37 | 3 | 47 | 78,72 | 46 | 4 | 0 | 50 |
| S281 | F | 19 | <=25 | 13 | <=14 | 37 | 37 | 3 | 47 | 78,72 | 42 | 8 | 9 | 41 |
| S333 | F | 21 | <=25 | 16 | >15 | 37 | 37 | 3 | 47 | 78,72 | 38 | 12 | 3 | 47 |
| S119 | M | 32 | >25 | 21 | >15 | 30 | 34 | 7 | 43 | 79,07 | 36 | 14 | 0 | 50 |
| S426 | M | 21 | <=25 | 11 | <=14 | 41 | 19 | 26 | 24 | 79,17 | 27 | 23 | 0 | 50 |
| S341 | F | 19 | <=25 | 14 | <=14 | 35 | 38 | 2 | 48 | 79,17 | 38 | 12 | 4 | 46 |
| S192 | F | 19 | <=25 | 13 | <=14 | 33 | 31 | 11 | 39 | 79,49 | 33 | 17 | 1 | 49 |
| S410 | F | 56 | >25 | 8 | <=14 | 48 | 35 | 6 | 44 | 79,55 | 40 | 10 | 4 | 46 |
| S099 | F | 20 | <=25 | 15 | >15 | 35 | 39 | 1 | 49 | 79,59 | 40 | 10 | 1 | 49 |
| S024 | F | 25 | <=25 | 16 | >15 | 41 | 39 | 1 | 49 | 79,59 | 40 | 10 | 1 | 49 |
| S018 | F | 30 | >25 | 16 | >15 | 41 | 39 | 1 | 49 | 79,59 | 48 | 2 | 5 | 45 |
| S030 | M | 32 | >25 | 21 | >15 | 34 | 39 | 1 | 49 | 79,59 | 40 | 10 | 2 | 48 |
| S249 | F | 19 | <=25 | 13 | <=14 | 28 | 40 | 0 | 50 | 80 | 46 | 4 | 4 | 46 |
| S290 | F | 19 | <=25 | 13 | <=14 | 28 | 20 | 25 | 25 | 80 | 26 | 24 | 5 | 45 |
| S304 | F | 19 | <=25 | 13 | <=14 | 36 | 36 | 5 | 45 | 80 | 43 | 7 | 24 | 26 |
| S045 | M | 24 | <=25 | 13 | <=14 | 36 | 40 | 0 | 50 | 80 | 43 | 7 | 0 | 50 |
| S148 | F | 27 | >25 | 13 | <=14 | 31 | 24 | 20 | 30 | 80 | 28 | 22 | 0 | 50 |
| S124 | F | 27 | >25 | 13 | <=14 | 44 | 36 | 5 | 45 | 80 | 43 | 7 | 2 | 48 |
| S202 | F | 22 | <=25 | 17 | >15 | 27 | 40 | 0 | 50 | 80 | 46 | 4 | 6 | 44 |
| S156 | F | 44 | >25 | 17 | >15 | 34 | 40 | 0 | 50 | 80 | 47 | 3 | 8 | 42 |
| S092 | M | 55 | >25 | 21 | >15 | 42 | 40 | 0 | 50 | 80 | 44 | 6 | 7 | 43 |
| S178 | F | 19 | <=25 | 13 | <=14 | 39 | 37 | 4 | 46 | 80,43 | 42 | 8 | 0 | 50 |
| S278 | M | 19 | <=25 | 13 | <=14 | 34 | 21 | 24 | 26 | 80,77 | 22 | 28 | 1 | 49 |
| S286 | F | 20 | <=25 | 13 | <=14 | 44 | 38 | 3 | 47 | 80,85 | 44 | 6 | 2 | 48 |
| S036 | F | 23 | <=25 | 13 | <=14 | 42 | 38 | 3 | 47 | 80,85 | 45 | 5 | 3 | 47 |
| S010 | F | 26 | >25 | 16 | >15 | 49 | 38 | 3 | 47 | 80,85 | 45 | 5 | 0 | 50 |
| S390 | M | 22 | <=25 | 17 | >15 | 38 | 38 | 3 | 47 | 80,85 | 42 | 8 | 3 | 47 |
| S179 | F | 23 | <=25 | 17 | >15 | 34 | 38 | 3 | 47 | 80,85 | 40 | 10 | 2 | 48 |
| S170 | F | 26 | >25 | 18 | >15 | 55 | 38 | 3 | 47 | 80,85 | 42 | 8 | 2 | 48 |
| S322 | F | 19 | <=25 | 14 | <=14 | 36 | 34 | 8 | 42 | 80,95 | 47 | 3 | 10 | 40 |
| S400 | F | 27 | >25 | 15 | >15 | 48 | 34 | 8 | 42 | 80,95 | 39 | 11 | 11 | 39 |
| S137 | F | 19 | <=25 | 14 | <=14 | 31 | 39 | 2 | 48 | 81,25 | 45 | 5 | 1 | 49 |
| S270 | M | 21 | <=25 | 13 | <=14 | 43 | 31 | 12 | 38 | 81,58 | 36 | 14 | 9 | 41 |
| S284 | F | 53 | >25 | 13 | <=14 | 33 | 40 | 1 | 49 | 81,63 | 47 | 3 | 9 | 41 |
| S007 | F | 27 | >25 | 16 | >15 | 39 | 40 | 1 | 49 | 81,63 | 49 | 1 | 5 | 45 |
| S180 | F | 23 | <=25 | 17 | >15 | 41 | 40 | 1 | 49 | 81,63 | 43 | 7 | 3 | 47 |
| S280 | F | 50 | >25 | 11 | <=14 | 41 | 36 | 6 | 44 | 81,82 | 41 | 9 | 0 | 50 |
| S066 | M | 36 | >25 | 16 | >15 | 31 | 41 | 0 | 50 | 82 | 44 | 6 | 2 | 48 |
| S386 | F | 32 | >25 | 17 | >15 | 25 | 41 | 0 | 50 | 82 | 43 | 7 | 0 | 50 |
| S078 | M | 27 | >25 | 18 | >15 | 51 | 41 | 0 | 50 | 82 | 46 | 4 | 3 | 47 |
| S231 | F | 20 | <=25 | 13 | <=14 | 39 | 37 | 5 | 45 | 82,22 | 39 | 11 | 2 | 48 |
| S035 | F | 54 | >25 | 13 | <=14 | 34 | 33 | 10 | 40 | 82,5 | 35 | 15 | 1 | 49 |
| S392 | M | 20 | <=25 | 14 | <=14 | 37 | 33 | 10 | 40 | 82,5 | 33 | 17 | 3 | 47 |
| S205 | F | 19 | <=25 | 8 | <=14 | 41 | 19 | 27 | 23 | 82,61 | 28 | 22 | 7 | 43 |
| S357 | F | 19 | <=25 | 13 | <=14 | 40 | 38 | 4 | 46 | 82,61 | 43 | 7 | 4 | 46 |
| S221 | F | 19 | <=25 | 13 | <=14 | 40 | 29 | 15 | 35 | 82,86 | 34 | 16 | 0 | 50 |
| S175 | M | 19 | <=25 | 13 | <=14 | 59 | 29 | 15 | 35 | 82,86 | 31 | 19 | 0 | 50 |
| S407 | F | 20 | <=25 | 14 | <=14 | 29 | 34 | 9 | 41 | 82,93 | 41 | 9 | 2 | 48 |
| S288 | F | 19 | <=25 | 13 | <=14 | 34 | 39 | 3 | 47 | 82,98 | 46 | 4 | 0 | 50 |
| S269 | F | 21 | <=25 | 16 | >15 | 38 | 39 | 3 | 47 | 82,98 | 41 | 9 | 3 | 47 |
| S061 | M | 55 | >25 | 12 | <=14 | 30 | 40 | 2 | 48 | 83,33 | 44 | 6 | 1 | 49 |
| S308 | F | 19 | <=25 | 13 | <=14 | 36 | 35 | 8 | 42 | 83,33 | 36 | 14 | 3 | 47 |
| S081 | M | 22 | <=25 | 13 | <=14 | 35 | 35 | 8 | 42 | 83,33 | 38 | 12 | 3 | 47 |
| S166 | F | 54 | >25 | 13 | <=14 | 64 | 40 | 2 | 48 | 83,33 | 42 | 8 | 0 | 50 |
| S374 | M | 23 | <=25 | 16 | >15 | 36 | 40 | 2 | 48 | 83,33 | 40 | 10 | 1 | 49 |
| S380 | F | 32 | >25 | 16 | >15 | 25 | 35 | 8 | 42 | 83,33 | 35 | 15 | 0 | 50 |
| S378 | F | 58 | >25 | 16 | >15 | 36 | 41 | 1 | 49 | 83,67 | 48 | 2 | 6 | 44 |
| S312 | F | 23 | <=25 | 13 | <=14 | 29 | 36 | 7 | 43 | 83,72 | 44 | 6 | 4 | 46 |
| S149 | F | 22 | <=25 | 18 | >15 | 53 | 36 | 7 | 43 | 83,72 | 42 | 8 | 0 | 50 |
| S076 | F | 27 | >25 | 18 | >15 | 31 | 36 | 7 | 43 | 83,72 | 43 | 7 | 1 | 49 |
| S075 | F | 19 | <=25 | 13 | <=14 | 33 | 42 | 0 | 50 | 84 | 45 | 5 | 1 | 49 |
| S242 | M | 52 | >25 | 13 | <=14 | 38 | 42 | 0 | 50 | 84 | 48 | 2 | 2 | 48 |
| S368 | F | 57 | >25 | 17 | >15 | 41 | 42 | 0 | 50 | 84 | 49 | 1 | 11 | 39 |
| S122 | F | 27 | >25 | 18 | >15 | 34 | 42 | 0 | 50 | 84 | 45 | 5 | 9 | 41 |
| S006 | M | 37 | >25 | 18 | >15 | 31 | 42 | 0 | 50 | 84 | 47 | 3 | 0 | 50 |
| S351 | F | 19 | <=25 | 14 | <=14 | 27 | 37 | 6 | 44 | 84,09 | 38 | 12 | 0 | 50 |
| S011 | F | 32 | >25 | 13 | <=14 | 41 | 38 | 5 | 45 | 84,44 | 48 | 2 | 2 | 48 |
| S343 | F | 23 | <=25 | 16 | >15 | 35 | 38 | 5 | 45 | 84,44 | 40 | 10 | 1 | 49 |
| S287 | M | 19 | <=25 | 13 | <=14 | 36 | 22 | 24 | 26 | 84,62 | 23 | 27 | 0 | 50 |
| S153 | F | 40 | >25 | 13 | <=14 | 48 | 39 | 4 | 46 | 84,78 | 43 | 7 | 0 | 50 |
| S332 | F | 19 | <=25 | 13 | <=14 | 31 | 34 | 10 | 40 | 85 | 37 | 13 | 0 | 50 |
| S244 | F | 58 | >25 | 8 | <=14 | 30 | 40 | 3 | 47 | 85,11 | 45 | 5 | 5 | 45 |
| S245 | M | 21 | <=25 | 12 | <=14 | 39 | 40 | 3 | 47 | 85,11 | 43 | 7 | 8 | 42 |
| S062 | F | 25 | <=25 | 13 | <=14 | 32 | 40 | 3 | 47 | 85,11 | 41 | 9 | 1 | 49 |
| S185 | F | 20 | <=25 | 14 | <=14 | 34 | 40 | 3 | 47 | 85,11 | 41 | 9 | 6 | 44 |
| S370 | F | 20 | <=25 | 14 | <=14 | 37 | 40 | 3 | 47 | 85,11 | 43 | 7 | 0 | 50 |
| S292 | F | 21 | <=25 | 13 | <=14 | 28 | 35 | 9 | 41 | 85,37 | 35 | 15 | 1 | 49 |
| S434 | M | 31 | >25 | 18 | >15 | 32 | 35 | 9 | 41 | 85,37 | 35 | 15 | 5 | 45 |
| S162 | F | 23 | <=25 | 11 | <=14 | 50 | 41 | 2 | 48 | 85,42 | 43 | 7 | 2 | 48 |
| S127 | F | 39 | >25 | 11 | <=14 | 29 | 41 | 2 | 48 | 85,42 | 47 | 3 | 13 | 37 |
| S191 | M | 20 | <=25 | 13 | <=14 | 39 | 41 | 2 | 48 | 85,42 | 45 | 5 | 1 | 49 |
| S087 | F | 19 | <=25 | 13 | <=14 | 40 | 42 | 1 | 49 | 85,71 | 45 | 5 | 2 | 48 |
| S373 | F | 20 | <=25 | 13 | <=14 | 49 | 42 | 1 | 49 | 85,71 | 44 | 6 | 12 | 38 |
| S057 | F | 34 | >25 | 17 | >15 | 57 | 42 | 1 | 49 | 85,71 | 43 | 7 | 1 | 49 |
| S097 | M | 58 | >25 | 17 | >15 | 62 | 43 | 0 | 50 | 86 | 48 | 2 | 3 | 47 |
| S047 | M | 36 | >25 | 18 | >15 | 33 | 43 | 0 | 50 | 86 | 47 | 3 | 8 | 42 |
| S237 | F | 22 | <=25 | 13 | <=14 | 24 | 38 | 6 | 44 | 86,36 | 39 | 11 | 0 | 50 |
| S084 | F | 26 | >25 | 18 | >15 | 36 | 34 | 11 | 39 | 87,18 | 39 | 11 | 1 | 49 |
| S182 | F | 19 | <=25 | 13 | <=14 | 36 | 41 | 3 | 47 | 87,23 | 44 | 6 | 1 | 49 |
| S360 | F | 48 | >25 | 13 | <=14 | 27 | 41 | 3 | 47 | 87,23 | 45 | 5 | 8 | 42 |
| S174 | F | 26 | >25 | 18 | >15 | 59 | 41 | 3 | 47 | 87,23 | 46 | 4 | 2 | 48 |
| S425 | M | 26 | >25 | 8 | <=14 | 39 | 42 | 2 | 48 | 87,5 | 47 | 3 | 11 | 39 |
| S140 | M | 43 | >25 | 13 | <=14 | 31 | 42 | 2 | 48 | 87,5 | 49 | 1 | 5 | 45 |
| S345 | F | 56 | >25 | 13 | <=14 | 81 | 42 | 2 | 48 | 87,5 | 44 | 6 | 1 | 49 |
| S145 | F | 31 | >25 | 17 | >15 | 36 | 43 | 1 | 49 | 87,76 | 44 | 6 | 0 | 50 |
| S111 | M | 45 | >25 | 17 | >15 | 46 | 43 | 1 | 49 | 87,76 | 48 | 2 | 7 | 43 |
| S133 | F | 26 | >25 | 18 | >15 | 28 | 43 | 1 | 49 | 87,76 | 44 | 6 | 4 | 46 |
| S017 | M | 30 | >25 | 18 | >15 | 31 | 43 | 1 | 49 | 87,76 | 44 | 6 | 1 | 49 |
| S022 | F | 38 | >25 | 18 | >15 | 32 | 43 | 1 | 49 | 87,76 | 45 | 5 | 1 | 49 |
| S044 | F | 31 | >25 | 21 | >15 | 45 | 43 | 1 | 49 | 87,76 | 45 | 5 | 0 | 50 |
| S272 | F | 31 | >25 | 23 | >15 | 44 | 43 | 1 | 49 | 87,76 | 46 | 4 | 1 | 49 |
| S190 | F | 19 | <=25 | 13 | <=14 | 34 | 36 | 9 | 41 | 87,8 | 41 | 9 | 0 | 50 |
| S159 | F | 27 | >25 | 21 | >15 | 24 | 36 | 9 | 41 | 87,8 | 45 | 5 | 0 | 50 |
| S402 | F | 49 | >25 | 8 | <=14 | 56 | 44 | 0 | 50 | 88 | 47 | 3 | 1 | 49 |
| S415 | M | 52 | >25 | 13 | <=14 | 68 | 44 | 0 | 50 | 88 | 43 | 7 | 2 | 48 |
| S325 | M | 58 | >25 | 13 | <=14 | 47 | 44 | 0 | 50 | 88 | 48 | 2 | 1 | 49 |
| S342 | M | 28 | >25 | 16 | >15 | 26 | 44 | 0 | 50 | 88 | 45 | 5 | 1 | 49 |
| S053 | F | 38 | >25 | 16 | >15 | 61 | 44 | 0 | 50 | 88 | 44 | 6 | 1 | 49 |
| S289 | F | 33 | >25 | 17 | >15 | 49 | 44 | 0 | 50 | 88 | 47 | 3 | 5 | 45 |
| S431 | F | 58 | >25 | 17 | >15 | 26 | 44 | 0 | 50 | 88 | 46 | 4 | 2 | 48 |
| S055 | F | 22 | <=25 | 18 | >15 | 63 | 44 | 0 | 50 | 88 | 45 | 5 | 1 | 49 |
| S003 | F | 24 | <=25 | 18 | >15 | 32 | 44 | 0 | 50 | 88 | 50 | 0 | 4 | 46 |
| S052 | F | 34 | >25 | 18 | >15 | 29 | 44 | 0 | 50 | 88 | 46 | 4 | 2 | 48 |
| S437 | F | 36 | >25 | 18 | >15 | 29 | 44 | 0 | 50 | 88 | 44 | 6 | 0 | 50 |
| S421 | M | 30 | >25 | 21 | >15 | 36 | 44 | 0 | 50 | 88 | 44 | 6 | 7 | 43 |
| S155 | F | 37 | >25 | 21 | >15 | 38 | 44 | 0 | 50 | 88 | 47 | 3 | 3 | 47 |
| S105 | F | 32 | >25 | 21 | >15 | 55 | 38 | 7 | 43 | 88,37 | 42 | 8 | 1 | 49 |
| S118 | F | 26 | >25 | 18 | >15 | 45 | 39 | 6 | 44 | 88,64 | 40 | 10 | 2 | 48 |
| S277 | F | 23 | <=25 | 17 | >15 | 32 | 40 | 5 | 45 | 88,89 | 40 | 10 | 1 | 49 |
| S416 | F | 56 | >25 | 17 | >15 | 28 | 40 | 5 | 45 | 88,89 | 32 | 18 | 3 | 47 |
| S065 | F | 28 | >25 | 16 | >15 | 54 | 41 | 4 | 46 | 89,13 | 44 | 6 | 0 | 50 |
| S423 | M | 43 | >25 | 13 | <=14 | 46 | 42 | 3 | 47 | 89,36 | 44 | 6 | 1 | 49 |
| S032 | F | 25 | <=25 | 16 | >15 | 35 | 34 | 12 | 38 | 89,47 | 39 | 11 | 1 | 49 |
| S210 | F | 18 | <=25 | 13 | <=14 | 29 | 43 | 2 | 48 | 89,58 | 43 | 7 | 8 | 42 |
| S216 | F | 19 | <=25 | 13 | <=14 | 32 | 43 | 2 | 48 | 89,58 | 44 | 6 | 0 | 50 |
| S388 | F | 25 | <=25 | 17 | >15 | 39 | 43 | 2 | 48 | 89,58 | 44 | 6 | 1 | 49 |
| S188 | F | 21 | <=25 | 14 | <=14 | 47 | 35 | 11 | 39 | 89,74 | 35 | 15 | 0 | 50 |
| S046 | M | 51 | >25 | 7 | <=14 | 31 | 44 | 1 | 49 | 89,8 | 46 | 4 | 0 | 50 |
| S177 | F | 21 | <=25 | 13 | <=14 | 54 | 44 | 1 | 49 | 89,8 | 46 | 4 | 0 | 50 |
| S419 | F | 56 | >25 | 13 | <=14 | 31 | 44 | 1 | 49 | 89,8 | 46 | 4 | 4 | 46 |
| S366 | F | 23 | <=25 | 18 | >15 | 32 | 44 | 1 | 49 | 89,8 | 44 | 6 | 4 | 46 |
| S123 | F | 32 | >25 | 21 | >15 | 28 | 44 | 1 | 49 | 89,8 | 45 | 5 | 7 | 43 |
| S169 | M | 36 | >25 | 12 | <=14 | 31 | 45 | 0 | 50 | 90 | 48 | 2 | 2 | 48 |
| S424 | M | 30 | >25 | 13 | <=14 | 35 | 45 | 0 | 50 | 90 | 50 | 0 | 4 | 46 |
| S014 | M | 39 | >25 | 13 | <=14 | 41 | 45 | 0 | 50 | 90 | 45 | 5 | 0 | 50 |
| S389 | M | 52 | >25 | 13 | <=14 | 39 | 45 | 0 | 50 | 90 | 48 | 2 | 2 | 48 |
| S404 | F | 26 | >25 | 16 | >15 | 29 | 45 | 0 | 50 | 90 | 45 | 5 | 4 | 46 |
| S054 | M | 30 | >25 | 16 | >15 | 40 | 45 | 0 | 50 | 90 | 45 | 5 | 1 | 49 |
| S167 | M | 37 | >25 | 17 | >15 | 46 | 45 | 0 | 50 | 90 | 48 | 2 | 1 | 49 |
| S121 | F | 27 | >25 | 18 | >15 | 31 | 45 | 0 | 50 | 90 | 47 | 3 | 4 | 46 |
| S109 | F | 30 | >25 | 18 | >15 | 34 | 45 | 0 | 50 | 90 | 46 | 4 | 1 | 49 |
| S117 | F | 32 | >25 | 18 | >15 | 29 | 45 | 0 | 50 | 90 | 45 | 5 | 3 | 47 |
| S417 | F | 34 | >25 | 18 | >15 | 37 | 45 | 0 | 50 | 90 | 46 | 4 | 0 | 50 |
| S019 | F | 38 | >25 | 18 | >15 | 22 | 45 | 0 | 50 | 90 | 48 | 2 | 2 | 48 |
| S016 | F | 38 | >25 | 18 | >15 | 74 | 45 | 0 | 50 | 90 | 47 | 3 | 2 | 48 |
| S114 | F | 28 | >25 | 21 | >15 | 40 | 45 | 0 | 50 | 90 | 45 | 5 | 1 | 49 |
| S152 | F | 45 | >25 | 21 | >15 | 37 | 45 | 0 | 50 | 90 | 45 | 5 | 0 | 50 |
| S094 | F | 27 | >25 | 18 | >15 | 39 | 37 | 9 | 41 | 90,24 | 39 | 11 | 0 | 50 |
| S364 | F | 19 | <=25 | 13 | <=14 | 67 | 39 | 7 | 43 | 90,7 | 45 | 5 | 1 | 49 |
| S161 | F | 22 | <=25 | 13 | <=14 | 56 | 40 | 6 | 44 | 90,91 | 44 | 6 | 1 | 49 |
| S235 | F | 20 | <=25 | 14 | <=14 | 46 | 40 | 6 | 44 | 90,91 | 40 | 10 | 1 | 49 |
| S329 | F | 46 | >25 | 10 | <=14 | 42 | 42 | 4 | 46 | 91,3 | 44 | 6 | 0 | 50 |
| S339 | F | 19 | <=25 | 14 | <=14 | 39 | 42 | 4 | 46 | 91,3 | 42 | 8 | 1 | 49 |
| S040 | F | 31 | >25 | 18 | >15 | 67 | 42 | 4 | 46 | 91,3 | 46 | 4 | 3 | 47 |
| S217 | F | 19 | <=25 | 13 | <=14 | 64 | 32 | 15 | 35 | 91,43 | 44 | 6 | 13 | 37 |
| S323 | F | 21 | <=25 | 10 | <=14 | 30 | 43 | 3 | 47 | 91,49 | 45 | 5 | 3 | 47 |
| S132 | M | 29 | >25 | 17 | >15 | 35 | 43 | 3 | 47 | 91,49 | 46 | 4 | 1 | 49 |
| S254 | M | 54 | >25 | 8 | <=14 | 32 | 44 | 2 | 48 | 91,67 | 44 | 6 | 1 | 49 |
| S408 | F | 43 | >25 | 21 | >15 | 42 | 44 | 2 | 48 | 91,67 | 46 | 4 | 1 | 49 |
| S048 | F | 36 | >25 | 18 | >15 | 32 | 45 | 1 | 49 | 91,84 | 45 | 5 | 0 | 50 |
| S436 | M | 40 | >25 | 18 | >15 | 38 | 45 | 1 | 49 | 91,84 | 45 | 5 | 0 | 50 |
| S193 | M | 19 | <=25 | 13 | <=14 | 38 | 34 | 13 | 37 | 91,89 | 41 | 9 | 4 | 46 |
| S098 | F | 23 | <=25 | 16 | >15 | 23 | 46 | 0 | 50 | 92 | 47 | 3 | 2 | 48 |
| S369 | F | 24 | <=25 | 16 | >15 | 36 | 46 | 0 | 50 | 92 | 48 | 2 | 11 | 39 |
| S232 | F | 27 | >25 | 18 | >15 | 38 | 46 | 0 | 50 | 92 | 46 | 4 | 2 | 48 |
| S093 | F | 29 | >25 | 18 | >15 | 47 | 46 | 0 | 50 | 92 | 48 | 2 | 0 | 50 |
| S013 | F | 40 | >25 | 18 | >15 | 35 | 46 | 0 | 50 | 92 | 47 | 3 | 0 | 50 |
| S091 | F | 34 | >25 | 21 | >15 | 39 | 46 | 0 | 50 | 92 | 47 | 3 | 8 | 42 |
| S086 | F | 38 | >25 | 21 | >15 | 36 | 46 | 0 | 50 | 92 | 46 | 4 | 3 | 47 |
| S196 | F | 19 | <=25 | 14 | <=14 | 40 | 35 | 12 | 38 | 92,11 | 36 | 14 | 1 | 49 |
| S042 | F | 26 | >25 | 13 | <=14 | 43 | 38 | 9 | 41 | 92,68 | 44 | 6 | 20 | 30 |
| S198 | M | 19 | <=25 | 13 | <=14 | 32 | 39 | 8 | 42 | 92,86 | 40 | 10 | 1 | 49 |
| S096 | F | 21 | <=25 | 13 | <=14 | 28 | 39 | 8 | 42 | 92,86 | 44 | 6 | 7 | 43 |
| S274 | F | 21 | <=25 | 16 | >15 | 26 | 41 | 6 | 44 | 93,18 | 44 | 6 | 0 | 50 |
| S228 | F | 53 | >25 | 21 | >15 | 28 | 42 | 5 | 45 | 93,33 | 43 | 7 | 1 | 49 |
| S387 | F | 23 | <=25 | 18 | >15 | 31 | 43 | 4 | 46 | 93,48 | 43 | 7 | 1 | 49 |
| S307 | F | 19 | <=25 | 13 | <=14 | 37 | 45 | 2 | 48 | 93,75 | 46 | 4 | 2 | 48 |
| S050 | M | 42 | >25 | 18 | >15 | 42 | 45 | 2 | 48 | 93,75 | 47 | 3 | 3 | 47 |
| S336 | M | 29 | >25 | 13 | <=14 | 31 | 47 | 0 | 50 | 94 | 49 | 1 | 1 | 49 |
| S257 | F | 44 | >25 | 13 | <=14 | 51 | 47 | 0 | 50 | 94 | 48 | 2 | 1 | 49 |
| S273 | F | 24 | <=25 | 15 | >15 | 33 | 47 | 0 | 50 | 94 | 47 | 3 | 7 | 43 |
| S313 | F | 22 | <=25 | 16 | >15 | 27 | 47 | 0 | 50 | 94 | 48 | 2 | 0 | 50 |
| S396 | F | 23 | <=25 | 18 | >15 | 28 | 47 | 0 | 50 | 94 | 47 | 3 | 0 | 50 |
| S012 | F | 28 | >25 | 18 | >15 | 23 | 47 | 0 | 50 | 94 | 49 | 1 | 2 | 48 |
| S128 | M | 29 | >25 | 18 | >15 | 29 | 47 | 0 | 50 | 94 | 47 | 3 | 5 | 45 |
| S120 | F | 30 | >25 | 18 | >15 | 39 | 47 | 0 | 50 | 94 | 49 | 1 | 0 | 50 |
| S164 | F | 34 | >25 | 18 | >15 | 27 | 47 | 0 | 50 | 94 | 47 | 3 | 2 | 48 |
| S112 | F | 40 | >25 | 18 | >15 | 32 | 47 | 0 | 50 | 94 | 49 | 1 | 1 | 49 |
| S147 | F | 41 | >25 | 18 | >15 | 36 | 47 | 0 | 50 | 94 | 47 | 3 | 2 | 48 |
| S319 | F | 57 | >25 | 18 | >15 | 37 | 47 | 0 | 50 | 94 | 47 | 3 | 11 | 39 |
| S130 | F | 31 | >25 | 21 | >15 | 56 | 47 | 0 | 50 | 94 | 48 | 2 | 1 | 49 |
| S385 | M | 38 | >25 | 21 | >15 | 37 | 47 | 0 | 50 | 94 | 47 | 3 | 1 | 49 |
| S315 | F | 19 | <=25 | 13 | <=14 | 30 | 34 | 14 | 36 | 94,44 | 39 | 11 | 1 | 49 |
| S340 | F | 24 | <=25 | 18 | >15 | 45 | 41 | 7 | 43 | 95,35 | 43 | 7 | 0 | 50 |
| S298 | F | 26 | >25 | 18 | >15 | 37 | 43 | 5 | 45 | 95,56 | 46 | 4 | 0 | 50 |
| S043 | M | 35 | >25 | 17 | >15 | 32 | 44 | 4 | 46 | 95,65 | 45 | 5 | 1 | 49 |
| S294 | F | 19 | <=25 | 8 | <=14 | 31 | 45 | 3 | 47 | 95,74 | 45 | 5 | 2 | 48 |
| S077 | M | 35 | >25 | 15 | >15 | 49 | 46 | 2 | 48 | 95,83 | 50 | 0 | 16 | 34 |
| S266 | F | 21 | <=25 | 16 | >15 | 34 | 47 | 1 | 49 | 95,92 | 49 | 1 | 0 | 50 |
| S422 | F | 23 | <=25 | 17 | >15 | 41 | 47 | 1 | 49 | 95,92 | 47 | 3 | 2 | 48 |
| S398 | F | 30 | >25 | 18 | >15 | 38 | 47 | 1 | 49 | 95,92 | 49 | 1 | 4 | 46 |
| S083 | M | 29 | >25 | 21 | >15 | 49 | 47 | 1 | 49 | 95,92 | 48 | 2 | 1 | 49 |
| S406 | F | 22 | <=25 | 13 | <=14 | 28 | 48 | 0 | 50 | 96 | 50 | 0 | 6 | 44 |
| S239 | F | 60 | >25 | 13 | <=14 | 42 | 48 | 0 | 50 | 96 | 48 | 2 | 0 | 50 |
| S027 | F | 27 | >25 | 16 | >15 | 29 | 48 | 0 | 50 | 96 | 48 | 2 | 2 | 48 |
| S113 | F | 28 | >25 | 18 | >15 | 37 | 48 | 0 | 50 | 96 | 48 | 2 | 15 | 35 |
| S420 | M | 30 | >25 | 18 | >15 | 37 | 48 | 0 | 50 | 96 | 48 | 2 | 0 | 50 |
| S321 | M | 28 | >25 | 20 | >15 | 33 | 48 | 0 | 50 | 96 | 49 | 1 | 2 | 48 |
| S253 | F | 20 | <=25 | 13 | <=14 | 46 | 43 | 6 | 44 | 97,73 | 44 | 6 | 1 | 49 |
| S354 | F | 19 | <=25 | 13 | <=14 | 32 | 45 | 4 | 46 | 97,83 | 47 | 3 | 1 | 49 |
| S101 | M | 31 | >25 | 21 | >15 | 38 | 47 | 2 | 48 | 97,92 | 47 | 3 | 1 | 49 |
| S233 | F | 19 | <=25 | 14 | <=14 | 36 | 48 | 1 | 49 | 97,96 | 49 | 1 | 7 | 43 |
| S151 | F | 41 | >25 | 21 | >15 | 26 | 48 | 1 | 49 | 97,96 | 47 | 3 | 3 | 47 |
| S362 | F | 23 | <=25 | 13 | <=14 | 49 | 49 | 0 | 50 | 98 | 50 | 0 | 15 | 35 |
| S206 | F | 20 | <=25 | 14 | <=14 | 31 | 49 | 0 | 50 | 98 | 49 | 1 | 1 | 49 |
| S034 | F | 26 | >25 | 16 | >15 | 30 | 49 | 0 | 50 | 98 | 49 | 1 | 5 | 45 |
| S051 | M | 27 | >25 | 16 | >15 | 37 | 49 | 0 | 50 | 98 | 49 | 1 | 0 | 50 |
| S173 | F | 31 | >25 | 16 | >15 | 28 | 49 | 0 | 50 | 98 | 49 | 1 | 0 | 50 |
| S082 | F | 25 | <=25 | 18 | >15 | 33 | 49 | 0 | 50 | 98 | 49 | 1 | 1 | 49 |
| S070 | F | 31 | >25 | 18 | >15 | 35 | 49 | 0 | 50 | 98 | 50 | 0 | 2 | 48 |
| S080 | F | 27 | >25 | 21 | >15 | 31 | 49 | 0 | 50 | 98 | 49 | 1 | 0 | 50 |
| S395 | F | 29 | >25 | 21 | >15 | 38 | 49 | 0 | 50 | 98 | 49 | 1 | 0 | 50 |
| S225 | F | 19 | <=25 | 13 | <=14 | 41 | 50 | 0 | 50 | 100 | 50 | 0 | 1 | 49 |
| S028 | F | 29 | >25 | 13 | <=14 | 25 | 50 | 0 | 50 | 100 | 50 | 0 | 0 | 50 |
| S125 | F | 34 | >25 | 13 | <=14 | 36 | 50 | 0 | 50 | 100 | 50 | 0 | 1 | 49 |
| S330 | F | 50 | >25 | 14 | <=14 | 27 | 49 | 1 | 49 | 100 | 49 | 1 | 0 | 50 |
| S384 | F | 21 | <=25 | 16 | >15 | 40 | 50 | 0 | 50 | 100 | 50 | 0 | 8 | 42 |
| S002 | F | 27 | >25 | 16 | >15 | 29 | 50 | 0 | 50 | 100 | 50 | 0 | 0 | 50 |
| S008 | M | 30 | >25 | 16 | >15 | 37 | 50 | 0 | 50 | 100 | 50 | 0 | 0 | 50 |
| S073 | M | 24 | <=25 | 18 | >15 | 40 | 50 | 0 | 50 | 100 | 50 | 0 | 3 | 47 |
| S079 | M | 27 | >25 | 18 | >15 | 39 | 50 | 0 | 50 | 100 | 50 | 0 | 0 | 50 |
| S074 | M | 31 | >25 | 18 | >15 | 27 | 50 | 0 | 50 | 100 | 50 | 0 | 0 | 50 |
| S297 | M | 48 | >25 | 18 | >15 | 31 | 50 | 0 | 50 | 100 | 50 | 0 | 0 | 50 |
| S102 | F | 38 | >25 | 22 | >15 | 27 | 50 | 0 | 50 | 100 | 50 | 0 | 0 | 50 |
|  |  |  |  |  |  |  |  |  |  |  |  |  |  |  |

**Supplementary Table 1**. Study dataset. ID: Identification code, Age_binary: age recoded following a median split procedure, School_binary: schooling years recoded following a median split procedure, PI-20: PI-20 total score, Recognized: absolute frequency of recognition across famous faces, Not Known in the checklist: absolute frequency of non-familiarity to the name (as assessed via the final checklist), Known in the checklist: absolute frequency of familiarity to the name (as assessed via the final checklist), Normalized accuracy: the absolute frequency of recognition divided by the frequency of recognition + frequency of non-recognition, Fam_HIT: hit on the familiarity toward a famous face, Fam_MISS: missing on the familiarity toward a famous face, Fam_FA: false alarm on the familiarity toward a non-famous face, Fam_CR: correct rejection on the familiarity toward a non-famous face.

| Age ≤ 25 years | |  | Age > 25 years | |
| --- | --- | --- | --- | --- |
| Raw Scores | T-Points |  | Raw Scores | T-Points |
| < 36 | < 23 |  | < 27 | < 19 |
| 36 | 23 |  | 27 | 19 |
| 37 | 24 |  | 28 | 20 |
| 38 | 24 |  | 29 | 21 |
| 39 | 25 |  | 30 | 21 |
| 40 | 25 |  | 31 | 22 |
| 41 | 26 |  | 32 | 23 |
| 42 | 30 |  | 33 | 23 |
| 43 | 31 |  | 34 | 24 |
| 44 | 31 |  | 35 | 25 |
| 45 | 32 |  | 36 | 25 |
| 46 | 33 |  | 37 | 26 |
| 47 | 35 |  | 38 | 26 |
| 48 | 35 |  | 39 | 27 |
| 49 | 36 |  | 40 | 28 |
| 50 | 36 |  | 41 | 29 |
| 51 | 37 |  | 42 | 30 |
| 52 | 37 |  | 43 | 30 |
| 53 | 37 |  | 44 | 31 |
| 54 | 37 |  | 45 | 31 |
| 55 | 37 |  | 46 | 31 |
| 56 | 38 |  | 47 | 32 |
| 57 | 39 |  | 48 | 32 |
| 58 | 39 |  | 49 | 33 |
| 59 | 40 |  | 50 | 34 |
| 60 | 40 |  | 51 | 34 |
| 61 | 40 |  | 52 | 34 |
| 62 | 41 |  | 53 | 35 |
| 63 | 41 |  | 54 | 35 |
| 64 | 42 |  | 55 | 35 |
| 65 | 43 |  | 56 | 35 |
| 66 | 43 |  | 57 | 36 |
| 67 | 43 |  | 58 | 36 |
| 68 | 44 |  | 59 | 36 |
| 69 | 45 |  | 60 | 36 |
| 70 | 45 |  | 61 | 37 |
| 71 | 46 |  | 62 | 37 |
| 72 | 47 |  | 63 | 37 |
| 73 | 48 |  | 64 | 38 |
| 74 | 48 |  | 65 | 38 |
| 75 | 49 |  | 66 | 38 |
| 76 | 49 |  | 67 | 39 |
| 77 | 50 |  | 68 | 40 |
| 78 | 51 |  | 69 | 40 |
| 79 | 52 |  | 70 | 41 |
| 80 | 53 |  | 71 | 42 |
| 81 | 53 |  | 72 | 42 |
| 82 | 54 |  | 73 | 43 |
| 83 | 55 |  | 74 | 44 |
| 84 | 56 |  | 75 | 44 |
| 85 | 57 |  | 76 | 45 |
| 86 | 58 |  | 77 | 45 |
| 87 | 59 |  | 78 | 46 |
| 88 | 59 |  | 79 | 46 |
| 89 | 59 |  | 80 | 46 |
| 90 | 60 |  | 81 | 47 |
| 91 | 61 |  | 82 | 47 |
| 92 | 62 |  | 83 | 48 |
| 93 | 63 |  | 84 | 49 |
| 94 | 64 |  | 85 | 50 |
| 95 | 66 |  | 86 | 50 |
| 96 | 66 |  | 87 | 50 |
| 97 | 67 |  | 88 | 51 |
| 98 | 68 |  | 89 | 54 |
| 99 | 71 |  | 90 | 54 |
| 100 | 74 |  | 91 | 57 |
|  |  |  | 92 | 57 |
|  |  |  | 93 | 59 |
|  |  |  | 94 | 59 |
|  |  |  | 95 | 60 |
|  |  |  | 96 | 62 |
|  |  |  | 97 | 63 |
|  |  |  | 98 | 64 |
|  |  |  | 99 | 66 |
|  |  |  | 100 | 68 |

**Supplementary Table 2**. Conversion of raw scores to T-Points scores of face recognition accuracy for young (≤ 25 years) and adult (> 25 years) samples, respectively. Differentiated scores for the two age groups were computed to account for the influence that age had in the ANOVA model.
